# Eclampsia

**Published:** 1903-03-07

**Authors:** 


### ECLAMPSIA.

THERE are certain morbid conditions which have up to the present supplied the purely speculative inquirer with happy hunting grounds enough, and possibly none has rejoiced him more than eclampsia. Until quite recently our friend, the arm-chair philosopher, has had matters all his own way in regard to this hitherto obscure disease, but, fortunately, owing to the exertions of many practical and careful investigators, important facts and observations have been steadily accumulating, and are even now sufficiently numerous and valuable to considerably narrow the field of research. Dr. Fothergill[1] is of opinion that the data are already sufficient to provide at any rate a sound working theory of the causation of the disease. He calls attention to a matter of some moment — the early diagnosis of the pre-eclamptic state and the prevention of eclampsia, which he states are now possible, and which illustrate the value of the latest ideas of the nature of this affection. He deals first with the modern theories and next with the modern treatment of eclampsia. The idea that it is a renal disease has been definitely abandoned, and it is generally considered now to be due to the presence in the circulating blood of a poison or poisons, the nature of which is unknown. The poison may be absorbed from the alimentary canal, or may originate in processes of metabolism. It is held that the pregnant woman is specially liable to intoxication because her organism has to deal not only with the waste products of her own tissue changes, but also with those due to the metabolism of the fœtus. Poisons circulating in the blood are dealt with by the liver, the kidneys, the thyroid and other glands, by which they are either rendered innocuous or expelled from the body. A breakdown on the part of any one of these "organs of defence" will throw the whole mechanism out of gear. Suppose the liver fails to neutralise a toxin circulating in the blood, the kidneys may be so injured by the poison that their excretive functions are diminished and the toxic agent accumulates in the system. Nicholson's theory of thyroid insufficiency being a cause of eclampsia is certainly plausible. Iodothyrin increases the excretion of urea. In eclampsia this is strikingly diminished. Owing to a deficiency of iodothyrin, it is thought, the metabolism of nitrogenous substances stops short of the formation of urea, and stops at a point when the products are toxic. In connection with this theory it should be remembered that the thyroid gland is enlarged in normal pregnancy, and that this enlargement is prevented by giving thyroid extract. Nicholson has reported cases of threatened eclampsia where the symptoms of toxæmia disappeared under thyroid treatment and returned repeatedly when the treatment was stopped. Dr. Fothergill places the measures for dealing with eclampsia under two heads, (1) prevention, (2) treatment of the actual eclamptic condition. Many of the minor ailments of pregnancy are symptoms of the pre-eclamptic state, such as vomiting, constipation, headache, nervous irritability, disturbances of vision, and abnormal pigmentation. The urine should be examined in all pregnancies from time to time, both for albumen and, quantitatively, for urea. The prophylactic treatment consists of rest in bed, milk diet, purgation, the administration of thyroid extract, and the washing out of the lower bowel with copious injections of warm water. Medical treatment is usually successful; if it fails, induction of labour is called for, whether the child be viable or not. Dealing with those cases in which convulsions come on before labour, Dr. Fothergill is of opinion that induction of labour will do more harm than good. In only mild cases can the patient survive both eclampsia and the shock of rapid artificial delivery; and in these mild cases medical treatment is usually sufficient. It consists of giving large doses of morphia, lavage of the lower bowel, the hot pack (if this is possible), and subcutaneous injections of saline solutions. Bleeding may be of service, and also the administration of thyroid extract. Since the treatment of eclampsia by copious subcutaneous injections of saline solution has been in use at the Glasgow Maternity Hospital the mortality of the disease at that institution has fallen from 47 per cent. to 17 per cent.

[1] Practitioner, February.

